# Emerging trends and focus on the link between gut microbiota and type 1 diabetes: A bibliometric and visualization analysis

**DOI:** 10.3389/fmicb.2023.1137595

**Published:** 2023-03-09

**Authors:** Keyu Guo, Jiaqi Li, Xia Li, Juan Huang, Zhiguang Zhou

**Affiliations:** ^1^National Clinical Research Center for Metabolic Diseases, Key Laboratory of Diabetes Immunology (Central South University), Ministry of Education, and Department of Metabolism and Endocrinology, The Second Xiangya Hospital of Central South University, Changsha, China; ^2^Section of Endocrinology, Department of Internal Medicine, School of Medicine, Yale University, New Haven, CT, United States

**Keywords:** type 1 diabetes, gut microbiota, research focus, bibliometric analysis, visualization analysis

## Abstract

**Objective:**

To conduct the first thorough bibliometric analysis to evaluate and quantify global research regarding to the gut microbiota and type 1 diabetes (T1D).

**Methods:**

A literature search for research studies on gut microbiota and T1D was conducted using the Web of Science Core Collection (WoSCC) database on 24 September 2022. VOSviewer software and the packages Bibliometrix R and ggplot used in RStudio were applied to perform the bibliometric and visualization analysis.

**Results:**

A total of 639 publications was extracted using the terms “gut microbiota” and “type 1 diabetes” (and their synonyms in MeSH). Ultimately, 324 articles were included in the bibliometric analysis. The United States and European countries are the main contributors to this field, and the top 10 most influential institutions are all based in the United States, Finland and Denmark. The three most influential researchers in this field are Li Wen, Jorma Ilonen and Mikael Knip. Historical direct citation analysis showed the evolution of the most cited papers in the field of T1D and gut microbiota. Clustering analysis defined seven clusters, covering the current main topics in both basic and clinical research on T1D and gut microbiota. The most commonly found high-frequency keywords in the period from 2018 to 2021 were “metagenomics,” “neutrophils” and “machine learning.”

**Conclusion:**

The application of multi-omics and machine learning approaches will be a necessary future step for better understanding gut microbiota in T1D. Finally, the future outlook for customized therapy toward reshaping gut microbiota of T1D patients remains promising.

## Introduction

Type 1 diabetes (T1D) is an autoimmune disease characterized by an ongoing destructive process due to aberrant antibodies and autoreactive T cell responses to self-antigens of pancreatic islet beta cells (β-cells), ultimately leading to absolute insulin deficiency (DiMeglio et al., 2018). Recently, an increasing number of studies have helped us understand how gut microbial dysbiosis disrupts immune homeostasis as a consequence of abnormal innate and adaptive immune responses to alterations in the gut microbiota and its metabolites, thereby engaging β-cell autoimmunity (Hu et al., 2015, 2017; Brown et al., 2019; Siljander et al., 2019).

Alterations in gut microbiota composition and intestinal permeability increase prior to T1D diagnosis, as shown by multiple large and well-characterized human T1D cohort studies (Davis-Richardson et al., 2014; Kemppainen et al., 2015; Kostic et al., 2015; Maffeis et al., 2016; Vatanen et al., 2016; Stewart et al., 2018; Vatanen et al., 2018; Harbison et al., 2019). Gut microbiota dysbiosis has also been observed in T1D patients (Brown et al., 2011; Mejía-León et al., 2014; Alkanani et al., 2015; Kostic et al., 2015; Stewart et al., 2018; Vatanen et al., 2018; Harbison et al., 2019; Huang et al., 2020a,b). Evidence from non-obese diabetic (NOD) mice, a widely used animal model of T1D, further confirmed that gut microbiota is involved in the progression of islet autoimmunity and T1D onset (Wen et al., 2008; Brown et al., 2016; Pearson et al., 2016; Huang et al., 2020a; Fuhri Snethlage et al., 2021; Huang et al., 2021; Girdhar et al., 2022; Jia et al., 2022; Marietta et al., 2022).

One of the critical T1D management strategies is to improve glycemic control and reduce complications in patients with T1D. More recently, research has focused on studying the association between gut microbiota and glycemic control and diabetic complications. The results showed that gut microbiota alteration is related to glycemic control, diabetic kidney disease, and macrovascular complications (Winther et al., 2020; van Heck et al., 2022; Shilo et al., 2022a). Based on these findings, therapeutic strategies targeting the gut microbiota were administrated in rodent and human studies to investigate their roles in preventing or reversing T1D progression, making remarkable advances (Mariño et al., 2017; Hänninen et al., 2018; Huang et al., 2020a; de Groot et al., 2021; Zou et al., 2021; Bell et al., 2022; He et al., 2022; Martens et al., 2022). Yet, despite these advances, further validation is required, and many challenges still need to be addressed.

Bibliometrics is a widely employed method to map studies within a specific research field by statistical and quantitative analysis of the academic impact and characteristics of publications. The aim is to complement empirical findings and to highlight frontier research and development trends toward guiding future directions in a rapidly evolving field such as gut microbiota. Global research trends in gut microbiota (Baudoin et al., 2019; Yuan et al., 2021; Zyoud et al., 2022a) and their roles in different diseases, including cancer (Zhang et al., 2020; Yang et al., 2022; Zyoud et al., 2022b), neuropsychiatric (Zyoud et al., 2019; Zhu et al., 2020; Cabanillas-Lazo et al., 2022; Wang H. et al., 2022; Zhao et al., 2022) and gastrointestinal disorders (Zyoud et al., 2021), among others, have been extensively explored and illustrated by bibliometric analysis.

Although previous research has studied the role of gut microbiota in T1D pathogenesis, diagnosis, prognostication, and treatment, to date no quantitative description of gut microbiota and T1D has been reported. In this article, we reviewed the role of gut microbiota in the field of T1D, identified related articles, and analyzed their characteristics. Our present study included research involving both animal models and human beings, allowing researchers to better understand how gut microbiota works in T1D.

## Methods

### Data source and search strategy

**Figure 1:**
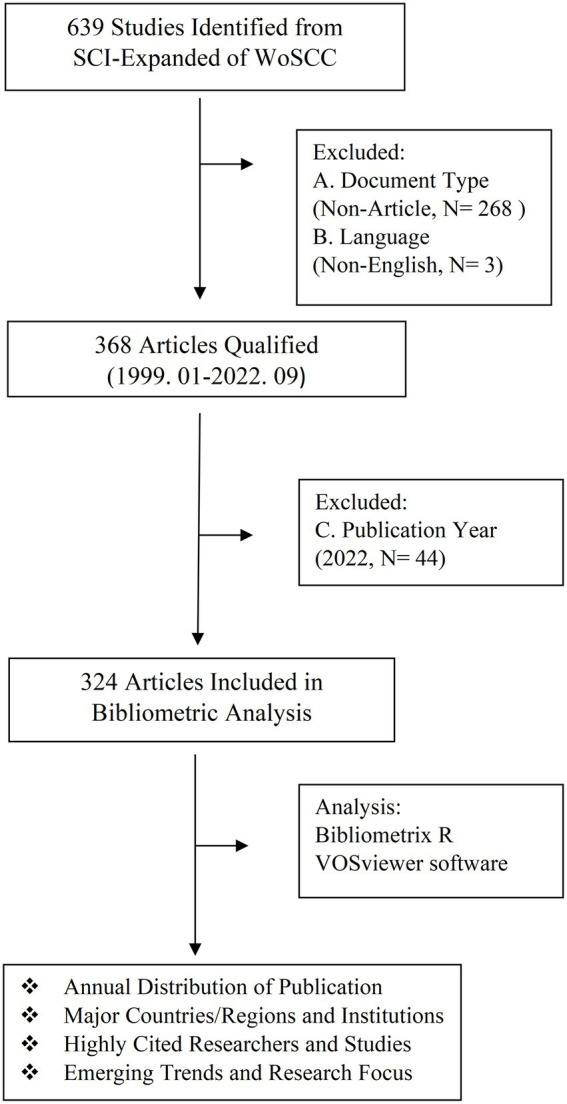
Workflow diagram of the literature search and screening of articles about gut microbiota and T1D.

The search strategy was set as TI = (type 1 diabetes and their synonyms) AND TI = (gut microbiota and their synonyms). All of these synonyms mainly refer to Medical Subject Headings (MeSHs) provided by the National Library of Medicine/PubMed, and the wildcard “*” was applied in place of any number of characters to identify as many relevant papers as possible. We searched Science Citation Index Expanded (SCIE) in Web of Science Core Collection (WoSCC) and screened all studies in this field published until 24 September 2022. The following exclusion criteria were used: (A) non-articles, such as conference abstracts and proceedings, corrigendum documents, retracted publication, letters, and edited materials, among others; (B) articles written in a language other than English; (C) articles with a publication date outside the time frame from 1 January 1999 to 31 December 2021. A total of 639 papers were retrieved from SCIE of WoSCC (Figure 1), and 324 articles were included in the following bibliometric and visualization analysis after screening.

### Bibliometric and visualize analysis

To perform bibliometric and visualization analysis, we used a Microsoft Excel 2019 (Microsoft, Redmond, WA, United States), the packages ggplot2 and Bibliometrix R in RStudio (version 2022.07.1, RStudio team, Boston, MA, United States), and VOSviewer software (version 1.6.18, Leiden University Science and Technology Research Center, the Netherlands).

## Results

### Annual distribution of publication

**Figure 2:**
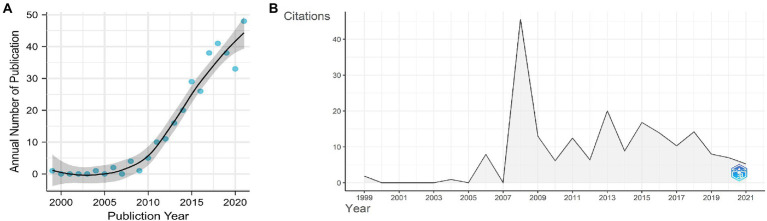
**(A)** Annual publication numbers and fit of the publication growth curve. **(B)** number of average citations per year.

From WoSCC, we retrieved 639 papers, among which 324 were original articles written in English from 49 countries/regions and published from 1999 to 2021. In the last two decades, the number of papers on gut microbiota and T1D indicates an upward trend (Figure 2A). Before 2008, only sporadic reports were available in the literature, but their numbers sharply increased thereafter, albeit not in the last 5 years, and only 44 articles were published in 2022 by the end of September.

The average annual citations of papers are shown in Figure 2B. The dynamic changes in average annual citations of articles may reflect that research from years with a high citation count had a significant academic impact. The whole distribution in average annual citations of papers showed six different peaks. The peak of annual citations that corresponds to the inflection point in the fitting curve is 2008. The graphical representation showed a dynamic variation of annual citations of papers on gut microbiota and T1D with a small peak every 2–3 years after 2008.

### Major countries/regions and institutions

**Figure 3:**
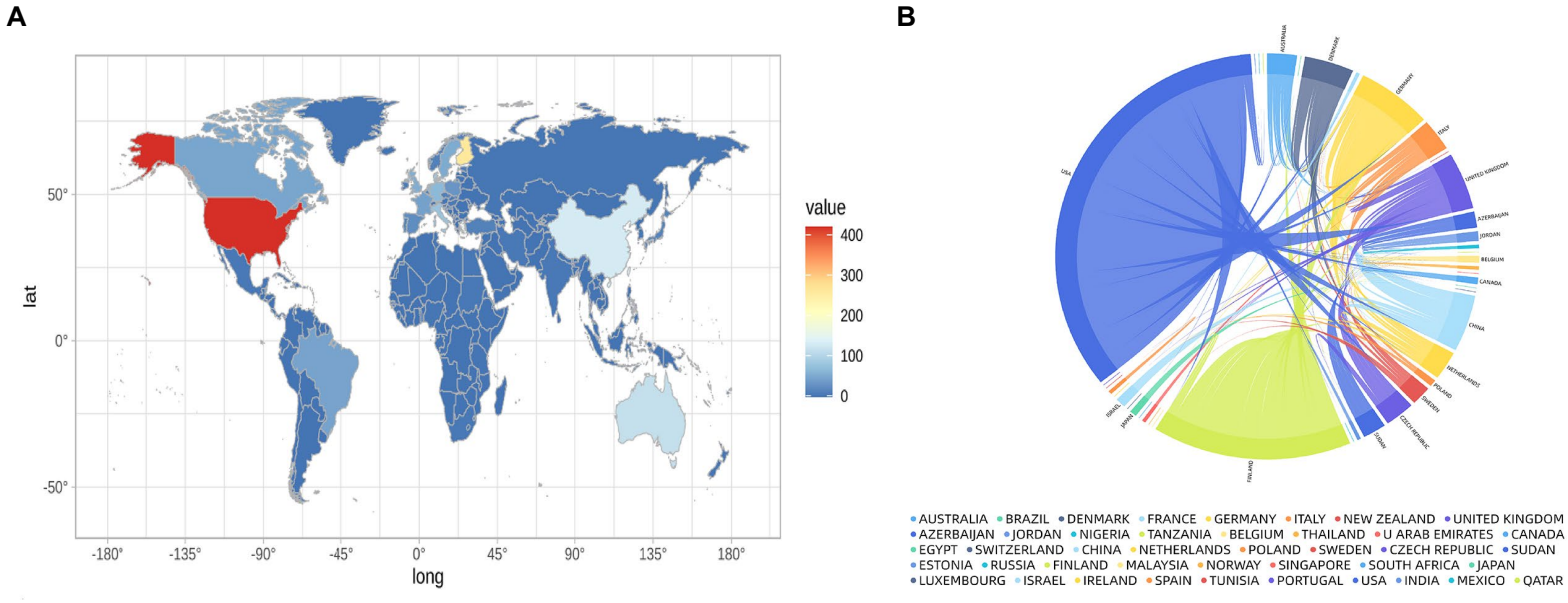
Distribution of countries conducting research and international collaborations in the field of gut microbiota and T1D. **(A)** World map showing the distribution of countries conducting research in this field. **(B)** International collaboration network of the top 20 countries.

**Figure 4:**
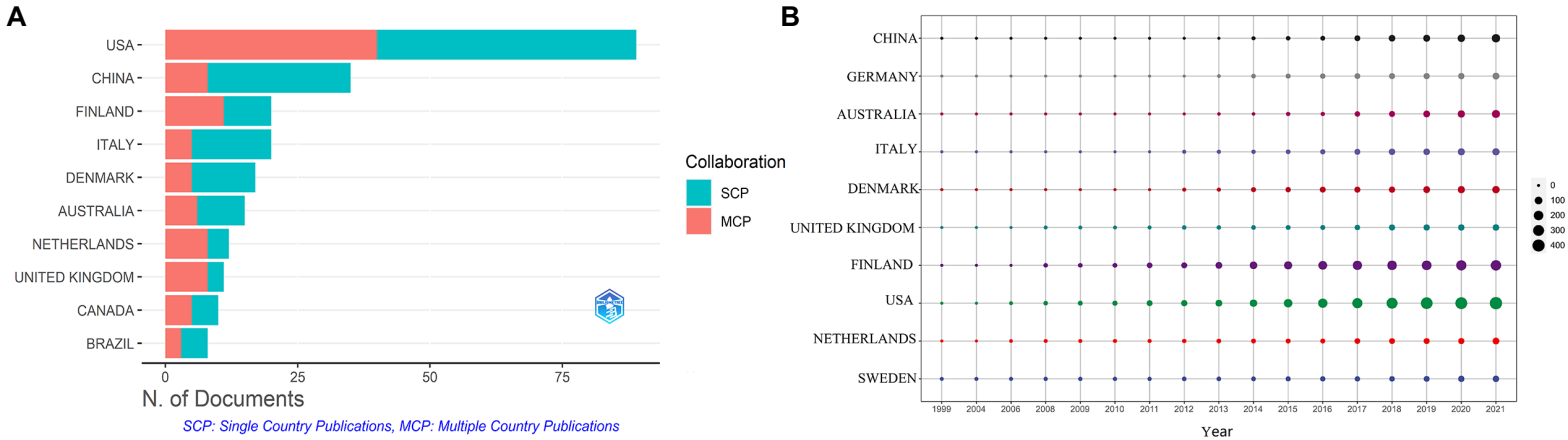
**(A)** Publication partnerships. **(B)** Annual contributions of the top 10 countries.

These studies originated from 49 different countries and regions, most of which (27.5%, *n* = 89) were from the United States, in addition to 35 studies from China, 20 from Finland, 20 from Italy, and 17 from Denmark. Most of these studies were designed and conducted by international teams and the frequency of occurrence of each coauthor’s country was visualized in the world map in Figure 3A. International collaboration networks of the top 20 countries were displayed in Figure 3B. The annual publications of the top 10 countries over time were shown in Figure 4. As shown in Figure 3, the United States not only accounts for most outputs but also is the center of international collaborations-most closely with European countries. The detailed quantitative analysis of collaboration papers of the top 10 countries also indicated that the publications of multiple countries/regions account for most of the worldwide research on gut microbiota and T1D (Figure 4). In addition, the top 10 most productive institutions and their annual publications are outlined in Table 1. The top 10 institutions are distributed as follows: six institutions in Finland (University of Helsinki, University of Turku, Tampere University Hospital, University of Oulu, Tampere University, and Turku University Hospital), three institutions in the United States (University of Florida, Yale University, and University of Colorado) and the University of Copenhagen in Denmark.

**Table 1:** Top 10 most productive institutions.

Rank	Institution	Country	NP
1	University of Florida	United States	56
2	University of Helsinki	Finland	50
3	University of Copenhagen	Denmark	43
4	University of Turku	Finland	41
5	Yale University	United States	27
6	Tampere University Hospital	Finland	23
7	University of Colorado	United States	22
8	University of Oulu	Finland	17
9	Tampere University	Finland	17
10	Turku University Hospital	Finland	16

NP, Number of papers; AC, Average citations per year; TC, Total citations.

### Highly cited researchers and studies

The top 10 most influential researchers are almost exclusively found in the top 10 most productive institutions. And the h-index, total citations, affiliations, and countries of the top 10 authors in number of publications were listed in Table 2 and the results showed that the top 10 authors are mainly from the United States and Finland. Table 3 showed that Li Wen had the highest number of publications, that Jorma Ilonen and Mikael Knip had the highest h-index, and that Mikael Knip had the most total citations. The top 10 authors not only are productive but also publish high-quality papers, and have a significant academic influence in the field of gut microbiota and T1D.

**Table 2:** Top 10 most productive countries/regions.

Rank	Country	TC	AC	NP
1	United States	8,590	96.52	89
2	Finland	1,649	82.45	20
3	Canada	1,542	154.20	10
4	United Kingdom	1,253	113.91	11
5	Denmark	1,018	59.88	17
6	China	805	23.00	35
7	Italy	795	39.75	20
8	Netherlands	784	65.33	12
9	Australia	748	49.87	15
10	Belgium	709	354.50	2

NP, Number of papers; AC, Average citations per year; TC, Total citations.

**Table 3:** Top 10 most influential researchers.

Element	NP	h-Index	TC	Institution	Country
Li Wen	16	12	1,997	Yale University	United States
Jorma Ilonen	15	13	2,912	University of Kuopio	Finland
Mikael Knip	15	13	2,966	University of Helsinki	Finland
F. Susan Wong	14	11	1,936	Cardiff University	England
Mark A. Atkinso	12	12	1,768	University of Florida	United States
Eric W. Triplett	11	11	1,522	University of Florida	United States
Heikki Hyöty	11	10	2,124	Tampere University	Finland
Olli Simell	11	10	1,505	Turku University Hospital	Finland
Anette-G Ziegler	11	10	1,499	Technical University Munich	Germany
Jian Peng	11	9	417	Yale University	United States

NP, Number of papers; TC, Total citations.

Furthermore, the 10 most cited papers outlined in Table 4, showing that Li Wen, who accounted for the largest share of the publications, had the most cited research in original articles on gut microbiota and T1D worldwide. These invaluable studies owned breakthrough discoveries, and the original articles were published in top-tier journals, such as *Nature*, *Science*, *Cell*, *Immunity*, and *New England Journal of Medicine* between 2008 and 2018.

**Table 4:** Global top 10 most cited papers.

Title	First author	Journal	Quartile	IF^*^	Year	TC	TC per year	Normalized TC
Innate immunity and intestinal microbiota in the development of type 1 diabetes	Li Wen	*Nature*	Q1	69.504	2008	1,375	91.67	2.16
Sex differences in the gut microbiome drive hormone-dependent regulation of autoimmunity	Janet G. M. Markle	*Science*	Q1	63.714	2013	1,109	110.90	6.16
How informative is the mouse for human gut microbiota research?	Thi Loan Anh Nguyen	*Dis Model Mech*	Q1	5.732	2015	687	85.88	5.85
Temporal development of the gut microbiome in early childhood from the teddy study	Christopher J. Stewart	*Nature*	Q1	69.504	2018	627	125.40	11.07
The dynamics of the human infant gut microbiome in development and in progression toward type 1 diabetes	Aleksandar D. Kostic	*Cell Host Microbe*	Q1	31.316	2015	612	76.50	5.21
Variation in microbiome LPS immunogenicity contributes to autoimmunity in humans	Tommi Vatanen	*Cell*	Q1	66.85	2016	555	79.29	6.67
Shared and distinct genetic variants in type 1 diabetes and celiac disease	Deborah J. Smyth	*New Engl J Med*	Q1	176.079	2008	517	34.47	0.81
Toward defining the autoimmune microbiome for type 1 diabetes	Adriana Giongo	*ISME J*	Q1	11.217	2011	516	43.00	3.78
Gender bias in autoimmunity is influenced by microbiota	Leonid Yurkovetskiy	*Immunity*	Q1	43.474	2013	516	51.60	2.87
Gut microbiota in children with type 1 diabetes differs from that in healthy children: a case–control study	Mora Murri	*BMC Med*	Q1	11.15	2013	443	44.30	2.46

* The impact factor (IF) and quartile of the journals refer to the 2021 Journal Citation Reports, TC, Total citations.

**Figure 5:**
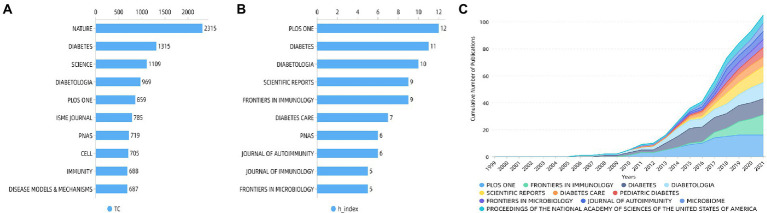
Top 10 journals by **(A)** total citations and by **(B)** h-index and **(C)** cumulative number of publications of the top 10 journals.

Total citation counts usually reflected the significance of the journal. The most cited journals were shown in Figure 5A, with *Nature* at the top of the list, followed by *Diabetes*, *Science*, *Diabetologia*, and *PLoS One*. In turn, the h-index expresses the academic influence of the journal, and *PLoS One* has the highest h-index followed by *Diabetes*, *Diabetologia*, *Scientific Reports*, and *Frontiers in immunology* (Figure 5B). Figure 5C summarized annual changes in the cumulative number of publications of these top 10 journals.

**Figure 6:**
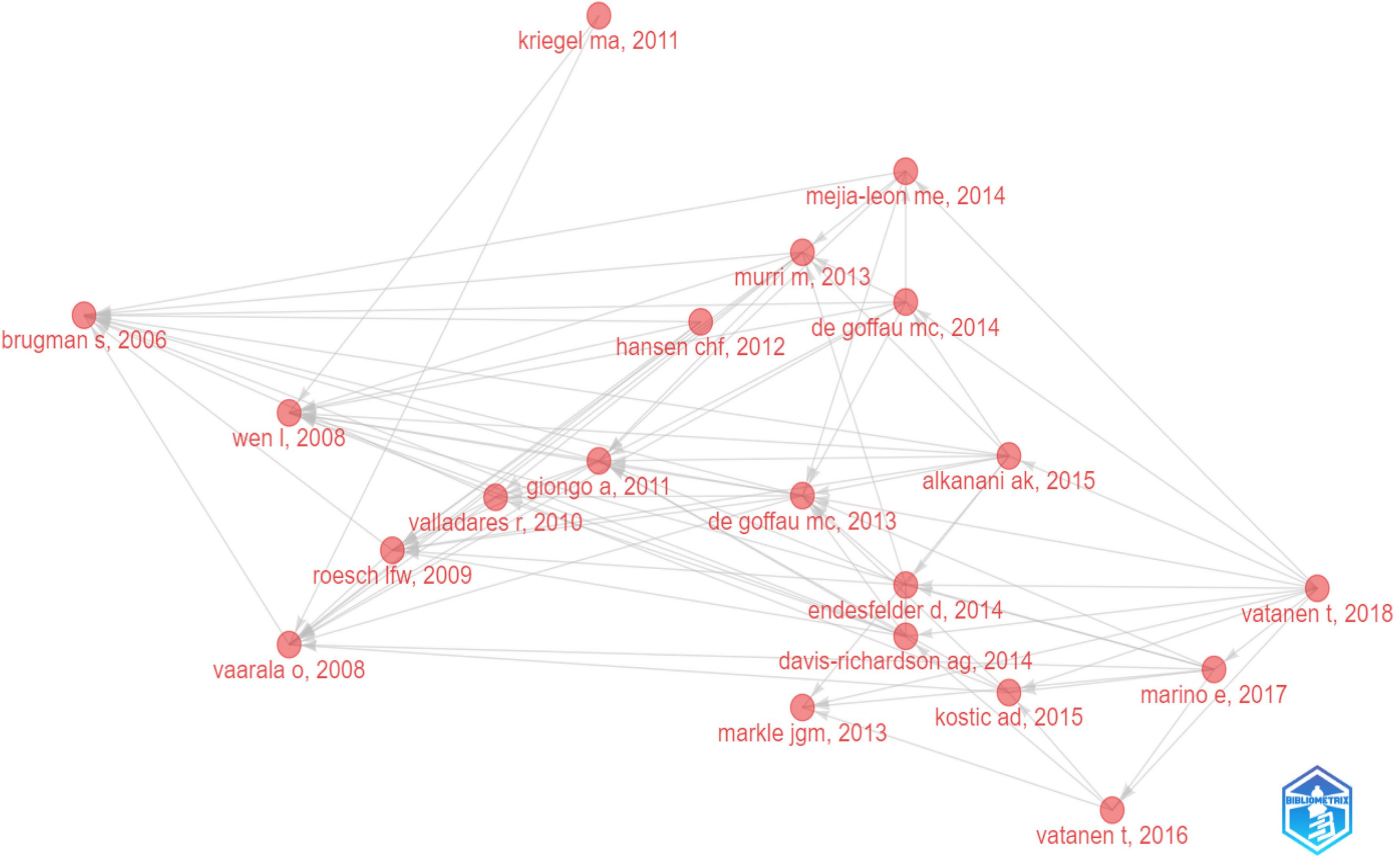
Historical direct citation network of seminal papers about gut microbiota and T1D.

The historical direct citation network of seminal papers on gut microbiota and T1D was shown in Figure 6, which was generated by historical cited paper visualization analysis. To further evaluate the quality of articles involved in historical direct network in the research, two metrics were applied, including the local citation score (LCS) which represented the sum number of citations of each specific article from the 324 publications included in bibliometric analysis, and the global citation score (GCS) which reflected the times an article has been cited by all documents in the WoSCC database (Table 5). It showed that the paper from Li Wen published in 2008 not only got on the topmost LCS but also won the highest GCS.

**Table 5:** Historical direct citation network of the publications.

Title	Journal	First author	Year	LCS	GCS
Antibiotic treatment partially protects against type 1 diabetes in the bio-breeding diabetes-prone rat	*Diabetologia*	Sylvia Brugman	2006	46	242
Innate immunity and intestinal microbiota in the development of type 1 diabetes	*Nature*	Li Wen	2008	111	1,375
The “perfect storm” for type 1 diabetes—the complex interplay between intestinal microbiota, gut permeability, and mucosal immunity	*Diabetes*	Outi Vaarala	2008	66	332
Culture-independent identification of gut bacteria correlated with the onset of diabetes in a rat model	*The ISME Journal*	Luiz F W Roesch	2009	39	168
*Lactobacillus Johnsonii* n6.2 mitigates the development of type 1 diabetes in BB-DP rats	*PLoS One*	Ricardo Valladares	2010	39	175
Toward defining the autoimmune microbiome for type 1 diabetes	*The ISME Journal*	Adriana Giongo	2011	104	516
Naturally transmitted segmented filamentous bacteria segregate with diabetes protection in nonobese diabetic mice	*P Natl Acad Sci USA*	Martin A. Kriegel	2011	32	310
Early life treatment with vancomycin propagates *Akkermansia muciniphila* and reduces diabetes incidence in the NOD mouse	*Diabetologia*	Camilla H. F. Hansen	2012	40	311
Gut microbiota in children with type 1 diabetes differs from that in healthy children: a case–control study	*BMC Med*	Mora Murri	2013	82	443
Fecal microbiota composition differs between children with beta-cell autoimmunity and those without	*Diabetes*	Marcus C. de Goffau	2013	84	349
Sex differences in the gut microbiome drive hormone-dependent regulation of autoimmunity	*Science*	Janet G. M. Markl	2013	44	1,109
Fecal microbiota imbalance in Mexican children with type 1 diabetes	*Sci Rep*	María Esther Mejía-León	2014	38	142
Aberrant gut microbiota composition at the onset of type 1 diabetes in young children	*Diabetologia*	Marcus C. de Goffau	2014	51	194
Compromised gut microbiota networks in children with anti-islet cell autoimmunity	*Diabetes*	David Endesfelder	2014	36	115
*Bacteroides dorei* dominates gut microbiome prior to autoimmunity in Finnish children at high risk for type 1 diabetes	*Front Microbiol*	Austin G. D. Richardson	2014	36	158
Alterations in intestinal microbiota correlate with susceptibility to type 1 diabetes	*Diabetes*	Aimon K. Alkanani	2015	28	155
The dynamics of the human infant gut microbiome in development and in progression toward type 1 diabetes	*Cell Host Microbe*	Aleksandar D. Kostic	2015	86	612
Variation in microbiome LPS immunogenicity contributes to autoimmunity in humans	*Cell*	Tommi Vatanen	2016	30	555
Gut microbial metabolites limit the frequency of autoimmune t cells and protect against type 1 diabetes	*Nat Immunol*	Eliana Mariño	2017	29	340
The human gut microbiome in early-onset type 1 diabetes from the teddy study	*Nature*	Tommi Vatanen	2018	34	313

LCS, Local citation score; GCS, Global citation score.

### Emerging trends and research focus

**Figure 7:**
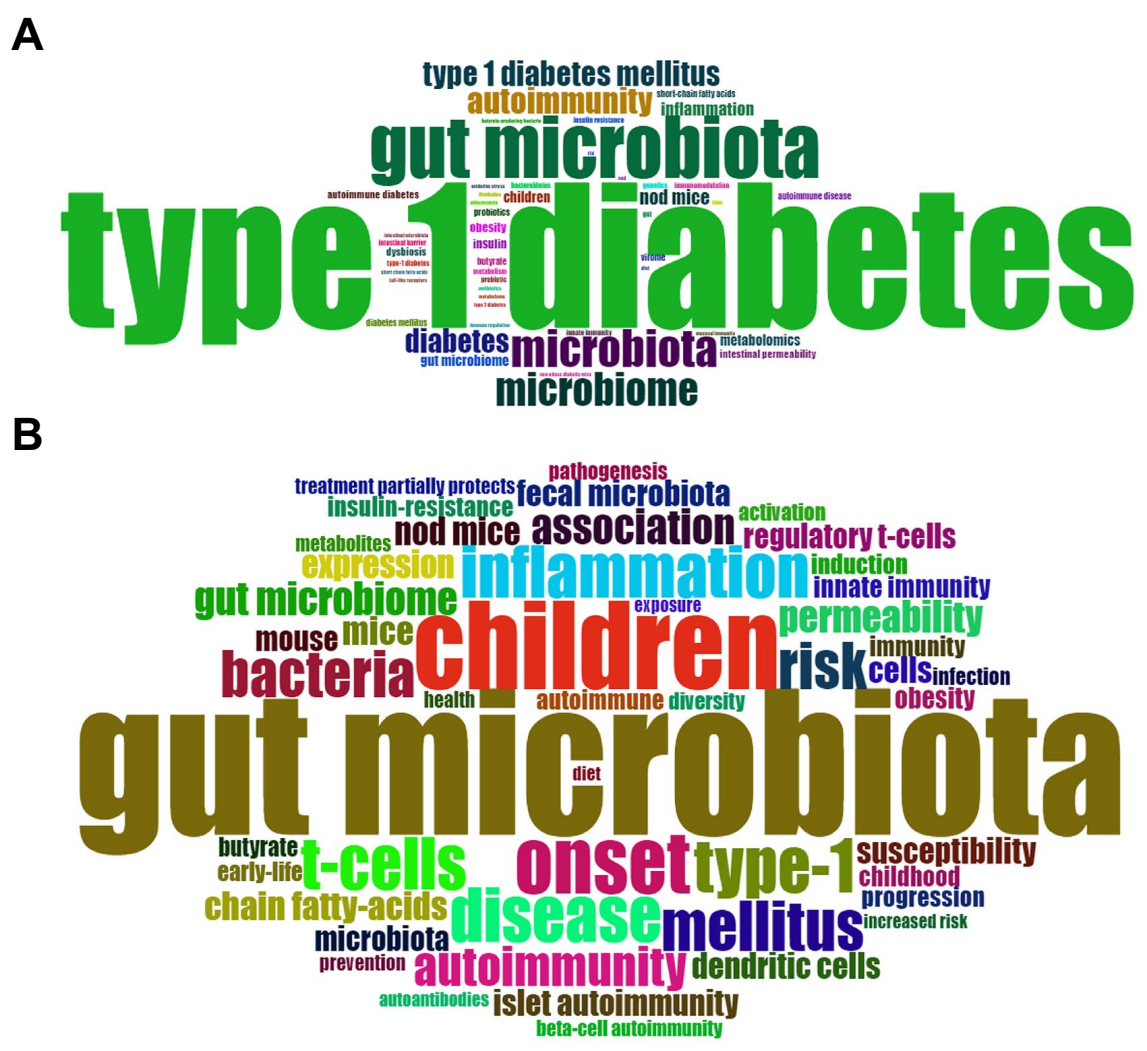
Distribution of the top 50 **(A)** Author Keywords and **(B)** Keywords Plus.

The keywords that describe the theme of the literature are important for highlighting hotspots in a specific research field. In present study, the top 50 most frequently used of author keywords and keywords plus were shown in a Word Cloud by visualization analysis (Figures 7A,B). The most frequently used author keyword was “autoimmunity” followed by “inflammation,” “children,” “metabolomics,” “obesity,” “dysbiosis,” “intestinal permeability,” “probiotics,” “short chain fatty acids,” “butyrate,” “virome,” “genetic,” and so forth. In addition, the most frequent keywords plus is “children,” followed by “inflammation,” “T-cell,” “autoimmunity,” “permeability,” “short chain fatty acids,” “regulatory T cell,” “innate immunity,” “obesity,” “insulin resistance,” “prevention,” and “diet.”

**Figure 8:**
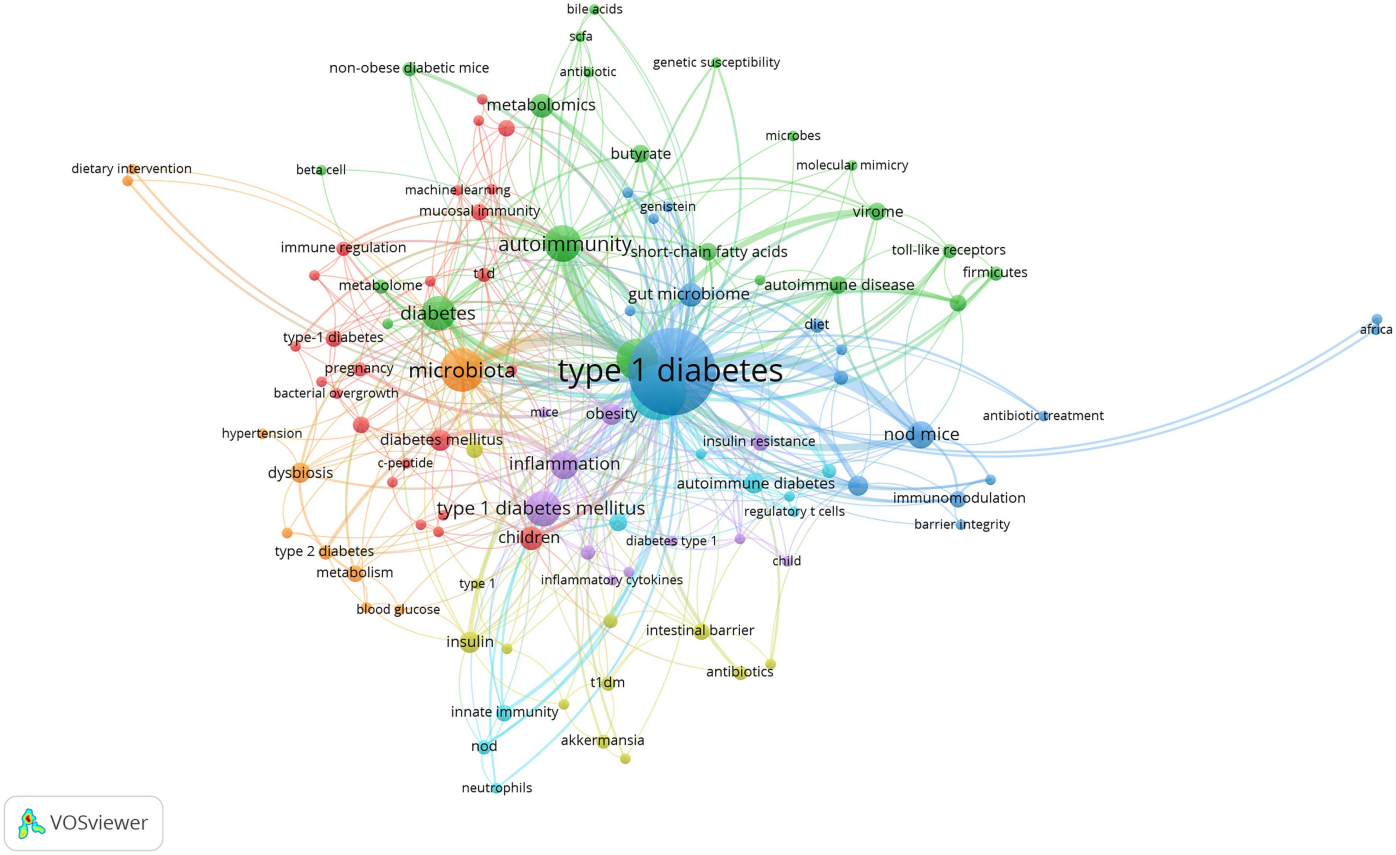
Cluster analysis of high-frequency keywords.

To further examine internal relationships between publications, clustering analysis was conducted based on all 106 keywords in VOSviewer (Figure 8). By total link strength, the 106 items were divided into seven clusters, and each cluster was highlighted with a specific color. Cluster 1 included 24 items, which were mainly correlated with epidemiology research on risk factors related to T1D onset (red). Cluster 2 consisted of 18 items, which mainly reflected the relationship between factors affecting gut microbiota and T1D (blue). Cluster 3 encompassed 22 items, which were associated with the relationship between alterations of the gut microbiome composition and metabolomics, and the role of molecular mimicry in β-cell autoimmunity (green). Cluster 4 comprised 12 items related to the relationship between the intestinal barrier and T1D onset (yellow). Cluster 5 contained 12 items, which reflected the inflammation and oxidative stress associated with gut microbiota involved in the metabolic disorder underlying T1D and T2D (purple). Cluster 6 covered 10 items, which were mainly related to the effect of the gut microbiome on immune responses in T1D (azure blue). Cluster 7 consisted of the last 10 items, which were mainly associated with therapeutic strategies targeting the gut microbiota in T1D (orange).

**Figure 9:**
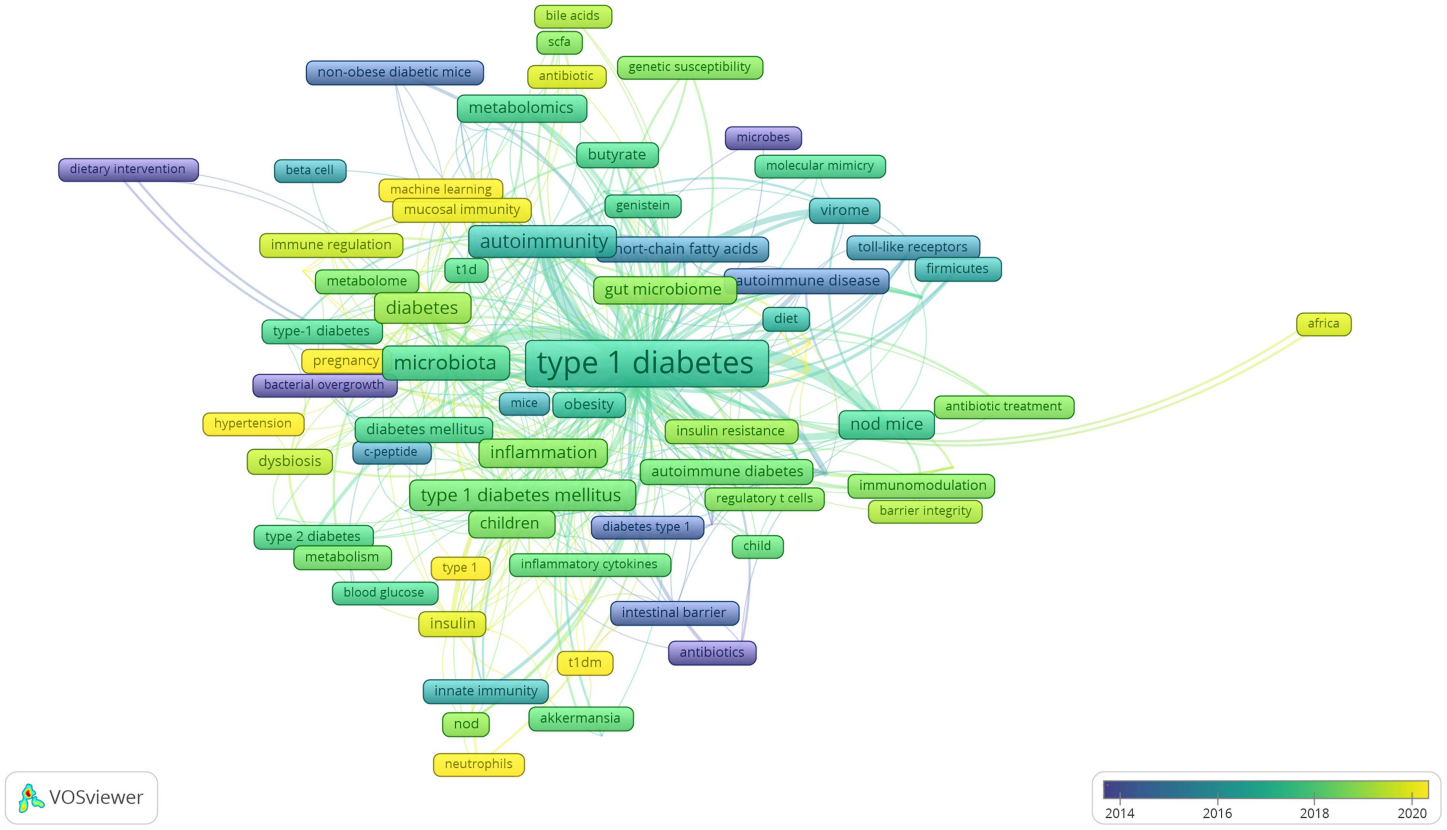
Developing trends of high-frequency keywords.

Lastly, we analyzed developing trends of high-frequency keywords of the gut microbiota and T1D research filed as the variation of keywords over time may express the evolution of hot topics and frontier research in this field and may have some significance in guiding future research. The visualization of high-frequency keywords over time was combined with year and frequency of keywords, and each keyword was mapped using different colors depending on the year. The closer the color was to blue, the earlier the keyword appeared, whereas the closer the color was to yellow, the more frequently the keywords appeared in recent published papers (Figure 9). The emergence of these seven clusters did not correlate with time, and the more yellow nodes were not found in a specific cluster, thus suggesting relatively balanced development dynamics in these clusters. The evolution of recent research hotspots can be compared by high-frequency keywords between the last 3 years and earlier stages. As shown in Figure 9, gut microbiota metabolites, such as “bile acids” and “short-chain fatty acids” are becoming increasingly popular. The topic about gut microbiota interaction with host immunity in T1D has been the focus of intense research, and “neutrophils” is an emerging keyword. Similarly, the relationship of gut microbiota with β-cell autoimmunity has remained an important topic for researchers to investigate in depth over time, but recent keywords such as “HbA1c,” “hyperglycemia,” “hypertension,” and “diabetic nephropathy” are increasingly more related to the clinical features and outcome of T1D.

Among keywords regarding countries and regions, “Asian” and “Africa” have appeared recently, indicating that more research has been conducted in these two regions. The results also showed that the “metagenomics” is more frequently applied than “16 s RNA sequencing” based on the use of sequencing technology keywords and that research has moved from “children” to “pregnancy” based on the life stage in which gut microbiota plays a role. Lastly, the emerging keyword “machine learning” has been an extension of traditional statistical methods and may become a valuable and increasingly necessary tool for exploring the relationship between gut microbiota and T1D.

## Discussion

The present study is the first bibliometric research about gut microbiota and T1D and includes a total of 324 articles retrieved from SCIE of WoSCC for bibliometric and visualization analysis. Since 2008, the number of publications per year has increased, and the average annual citations of articles also peaked in 2008. Based on the fit of the curve and actual data of 2022, we project that the number of publications per year will remain at relatively constant level for a period.

Four publications in 2008 were included in our analysis, and subsequent analysis of the historical citation network suggested that a study conducted by Li Wen and her colleagues is the most cited original article and a milestone in the field of research on gut microbiota and T1D. In that study, Li Wen et al. found that Myd88 deficiency protects NOD mice from T1D, whereas Myd88^−/−^ NOD mice under germ-free conditions did develop T1D (Wen et al., 2008). Their study revealed for the first time that the interplay between the host innate immune system and gut microbiota is at the root of T1D pathogenesis, thus laying a solid foundation for future research.

European countries and the United States are the leading contributors to this field. Research success in those countries may be linked to their well-characterized cohort studies, the most representatives of which are The Environmental Determinants of Diabetes in the Young (TEDDY), the Pathogenesis of Type 1 Diabetes-Testing the Hygiene Hypothesis (DIABIMMUNE), and All Babies in Southeast Sweden (ABIS) studies. To identify environmental factors related to the onset of T1D, the TEDDY study recruited newborns with high-risk human leukocyte antigen (HLA) alleles from the general population and first-degree relatives (FDRs) of T1D patients, who were prospectively analyzed and followed up at different clinical centers, including centers in Colorado, Washington State, Georgia, Florida in the United States, centers in Finland, Germany and Sweden. DIABIMMUNE is another important multinational longitudinal study for testing the hygiene hypothesis in T1D, which recruited newborns with high-risk HLA haplotypes from Finland, Estonia, and Russian Karelia. In addition, ABIS from Sweden is a prospective birth cohort aimed at examining the role of gut microbiota in the etiology of T1D. Diet intervention studies, such as the Primary dietary intervention study to reduce the risk of islet autoimmunity in children at increased risk for type 1 diabetes (BABYDIET), the Finnish Diabetes Prediction and Prevention Project (DIPP), and the Trial to Reduce IDDM in the Genetically at Risk (TRIGR) also directly or indirectly addressed the association between gut microbiota and islet β-cell autoimmunity.

Moreover, we relied on animal models to explore how gut microbiota regulate T1D pathogenesis (Tlaskalová-Hogenová et al., 2011; Pearson et al., 2016). Evidence has shown that NOD mice develop spontaneous β-cell autoimmunity mimicking T1D and the development of this animal model under specific pathogen free and germ free condition enables research on gut microbiota to move forward from bedside to bench. Although the current main researchers and research institutions are located in the United States and European countries, the keywords “Asian” and “Africa” have appeared recently, indicating that research on T1D and gut microbiota is starting to gain widespread attention in these two regions. To compensate for research disadvantages in Asian and Africa in relevant animal studies, such as the later start, the lack of depth of research, the relatively small size of human studies, and most studies are cross-sectional. Therefore, actions to improve research conditions and techniques, in addition to setting up large well-characterized cohorts in Asian and Africa is needed.

In the present study, we analyzed publications on gut microbiota and T1D and listed the most influential research, researchers, and institutions. This information may be helpful for those who want to learn from the outstanding studies and seek collaboration opportunities. Another key feature of our study consisted of highlighting the hotspots and change trends of research on T1D and gut microbiota. The high-frequency keywords and clustering analysis indicated the current hotspots and main research directions. Our analysis of animal studies and human research conducted in the past two decades suggested that gut microbiota is closely related to the onset of islet β-cell autoimmunity in T1D from seven perspectives. Several environmental triggers, especially gut microbiota, contribute to islet β-cell autoimmunity based on human research (Cluster 1 and Cluster 2), and the most prevalent stage has advanced from children to early pregnancy although the exact reasons for this shift must be further investigated. Subsequently, the causative link between gut microbiota and T1D has gradually been proved in animal models. From Cluster 3 to Cluster 6, the hot topics explored by researchers are associated with the mechanism underlying the effect of gut microbiota on T1D, including alterations in the gut microbiota and its metabolites, which can cause changes in intestinal permeability, mimic molecules in β-cell islets, and induce oxidative stress, inflammation abnormal innate and adaptive immune responses.

Among the top of the 50 most frequently used author keywords and keywords plus, 16 s RNA sequencing and detecting short chain fatty acids stand out as major methods for exploring alterations in the gut microbiome composition and its metabolites. The terms “metagenomics” and “metabolomics” have been more frequently used in recent research, and the advent of multi-omics profiling technologies has enabled us to identify the gut microbiome composition at species level, to explore the functional potential of the gut microbiome, and to investigate mycobiomes and virome in to the context of T1D (Vehik et al., 2019; Auchtung et al., 2022). These new research technologies are more comprehensive and accurate tools for testing and verifying alterations in the gut microbiota and its effect on T1D.

The keywords cloud has showed that T cells play a key role in T1D progression regulated by gut microbiota, as confirmed by many studies and this research continues. Moreover, other components of the immune system, most recently neutrophils, have also gained considerable research attention. Some of the important goals of these studies are translational applications of gut microbiota toward improving therapeutic strategies for T1D (Cluster 7). Recently, an increasing number of studies have suggested that gut microbiota is involved in the glycemic control and complications of T1D. Therefore, gut microbiota-targeted therapeutic strategies are promising new approaches not only in preventing or halting disease progression but also in improving the clinical outcome of T1D patients. The present gut microbiota-targeted therapeutic strategy in T1D, which consists of diet, probiotics, prebiotics, fecal transplant, among other measures, has made only an initial progress. However, gut microbiota-targeted therapeutic strategies have already brought venture, benefits, challenges, and opportunities for the prevention and cure of T1D.

Machine learning, a newly emerged keyword in the field of gut microbiota in T1D, has promoted the development of research on integrating gut microbiota to predict disease progress and therapeutic response. Current research has revealed that gut microbiota may be able to predict the diabetes risk. Simultaneously, whether in individuals without diabetes or patients with type 2 diabetes (T2D) or T1D, gut microbiota can predict the individual postprandial glucose response (Zeevi et al., 2015; Mendes-Soares et al., 2019; Shilo et al., 2022b). In addition, integrating clinical and gut microbiome characteristics can predict the short-and long-term glucose status and β-cell function of T2D patients (Aasmets et al., 2021). In the field of T1D, the microbial risk score and the genetic risk score model predicted well the impact of interactions between host genetics and gut microbiome on disease progression (Wang C. et al., 2022). We believe that machine learning is a potential research direction in the field of gut microbiota and T1D, which enables the integration of medical information and gut microbiota data to identify predictive microbiota biomarkers and to develop customized therapy toward reshaping the gut microbiota of T1D patients.

## Conclusion

In the present study, we extracted the publications of original research articles on gut microbiota and T1D published in SCIE of WoSCC and conducted bibliometric and visualization analysis. The development and deep research on gut microbiota in NOD mice would bringing T1D to a new translational level in the field of gut microbiota. And the application of multi-omics and machine learning approaches will be a necessary future step for better understanding the gut microbiota in T1D and develop customized therapy toward reshaping the gut microbiota of T1D patients. Based on these, the warming of gut microbiota-targeted therapeutic strategies that would help develop optimal strategies for “precision” gut microbiota modulation in T1D, and progress is being made toward this goal, but there is still a lot to do.

## Data availability statement

The original contributions presented in the study are included in the article/supplementary material, further inquiries can be directed to the corresponding authors.

## Author contributions

KG, JL, and JH performed the literature search and drafted the manuscript. JH provided ideas and suggestions. KG, JL, XL, JH, and ZZ discussed and revised the manuscript. All authors contributed to the article and approved the submitted version.

## Funding

This work was funded by the National Natural Science Foundation of China (grant nos. 82100899 awarded to JH and 81820108007 awarded to ZZ), the Natural Science Foundation of the Hunan province of China (grant no. 2021JJ40833 awarded to JH) and the Pilot and Feasibility grant of the Yale Diabetes Research Center (grant no. DK 045735 awarded to JH).

## Conflict of interest

The authors declare that the research was conducted in the absence of any commercial or financial relationships that could be construed as a potential conflict of interest.

## Publisher’s note

All claims expressed in this article are solely those of the authors and do not necessarily represent those of their affiliated organizations, or those of the publisher, the editors and the reviewers. Any product that may be evaluated in this article, or claim that may be made by its manufacturer, is not guaranteed or endorsed by the publisher.
